# COVID-19—A Theory of Autoimmunity Against ACE-2 Explained

**DOI:** 10.3389/fimmu.2021.582166

**Published:** 2021-03-23

**Authors:** Philip McMillan, Thomas Dexhiemer, Richard R. Neubig, Bruce D. Uhal

**Affiliations:** ^1^Doncaster and Bassetlaw National Health Service (NHS) Trust, Doncaster, United Kingdom; ^2^Department of Pharmacology and Toxicology, Michigan State University, East Lansing, MI, United States; ^3^Nicholas V. Perricone, M.D., Division of Dermatology, Department of Medicine, Michigan State University, East Lansing, MI, United States; ^4^Department of Physiology, Michigan State University, East Lansing, MI, United States

**Keywords:** COVID-19, autoimmunity, lung, solubleACE-2, macrophage

## Abstract

The COVID-19 pandemic caused by the coronavirus SARS-COV-2 has cost many lives worldwide. In dealing with affected patients, the physician is faced with a very unusual pattern of organ damage that is not easily explained on the basis of prior knowledge of viral-induced pathogenesis. It is established that the main receptor for viral entry into tissues is the protein angiotensin-converting enzyme-2 [“ACE-2”, (1)]. In a recent publication (2), a theory of autoimmunity against ACE-2, and/or against the ACE-2/SARS-COV-2 spike protein complex or degradation products thereof, was proposed as a possible explanation for the unusual pattern of organ damage seen in COVID-19. In the light of more recent information, this manuscript expands on the earlier proposed theory and offers additional, testable hypotheses that could explain both the pattern and timeline of organ dysfunction most often observed in COVID-19.

## Background of COVID-19

The first epidemic involving Severe Acute Respiratory Syndrome (“SARS”) was identified in 2003 as a novel clinical entity (3). This SARS-COV infection was primarily clustered in Asia (4) with some international spread, and ~770 deaths and over 8,000 people affected worldwide (5). SARS-COV belongs to the family *Coronaviridae*, which comprises enveloped RNA viruses in the order Nidovirales (6), and is the largest known non-segmented genome among RNA viruses (7) with ~15 spike proteins on the surface of each virion (8). These spike proteins represent the attachment point for entry of the virus into the cell. The reason for the disappearance of this dangerous infection is unclear, but effective preventative measures and an antigenic shift may be possible explanations (9).

The current COVID-19 pandemic was triggered by the spread of a novel coronavirus SARS-COV-2 (10), with the earliest reports coming out of Wuhan, China, in late 2019. Although it has an 80% similarity to SARS-COV, there are specific differences within the receptor-binding domain of the spike protein which impact on infectivity (1, 11). This variation has a combination of high infectivity and spread through undocumented infection, promoting rapid dissemination across the world (12). The severe organ damage, which occurs in a subset of people affected by the virus, is similar to that caused by SARS-COV, but occurs on a much larger scale because of the higher number of people infected. People at risk of severe disease represent ~2.3% of those who contract the virus, and are primarily the elderly and those with comorbidities (13).

## Current Understanding of the Disease

ACE-2 is a Type 1 membrane-bound glycoprotein with 42% homology to angiotensin-converting enzyme (“ACE”) (14). The primary purpose of ACE-2 is to convert angiotensin II to angiotensin (1–7) which has an impact on arterial pressures and inflammation (15). This role is counter regulatory to ACE and serves to balance the renin-angiotensin system (“RAS”). It is relevant in COVID-19 because ACE-2 is also the entry receptor for SARS-COV-2.

The current thinking is that SARS-COV-2 targets the ACE-2 entry receptor (16), causing direct viral cellular damage, with the release of excessive immune mediators and coagulation abnormalities (17). Identification of viral particles in the lung tissue supports this mechanism for damage (18). Release of immune factors (19) appears to be part of the trigger for an associated cytokine storm. Elevated levels of interleukins, tumor necrosis factor and interferons have been observed (20). Immune-mediated disseminated intravascular coagulation with lung micro-thrombosis is an observed pattern across patients (21, 22). There is also the perspective that damage to the ACE-2 receptor could impact on the ability of RAS to function effectively (23). The presence of ACE-2 receptors in the kidney may offer an explanation of the extent of renal dysfunction in more severely affected patients (24).

These hypotheses are, however, unable to explain the severity of the disease and involvement of multiple organs with vasculitic-type responses in SARS-COV-2.

## What Is Soluble ACE-2?

Soluble ACE-2, also called serum or plasma ACE-2, refers to the ACE-2 enzyme ectodomain that has been cleaved from the cell surface, a process called shedding (25). The purpose of shedding is unclear (26), but it seems to occur more frequently in hypertension and heart disease (27). Clinically relevant stimuli, such as supplemental oxygen at levels routinely used in neonatal medicine (FiO2 0.95), have also been shown to result in ACE-2 shedding from human lung cells in culture (28). This process reduces the amount of ACE-2 localized to the cells, but increases that free to enter other tissue compartments. To date, the functions and fate(s) of this soluble ACE-2 in different organs has not been rigorously studied.

Soluble ACE-2 has been shown to bind efficiently to the spike protein of SARS-COV (29) and, although not confirmed by direct measurements as of the time of this writing, such binding is assumed to occur with SARS-COV-2 as well. Indeed, recent modeling studies based on the sequence of soluble ACE-2 and the SARS-CoV2 spike protein (30) strongly predicted that SARS-CoV-2 can not only bind to soluble ACE-2, but also suggest that the strength of binding might be negatively impacted by nicotine, a finding which is being considered for potential therapeutic value.

**Figure 1:**
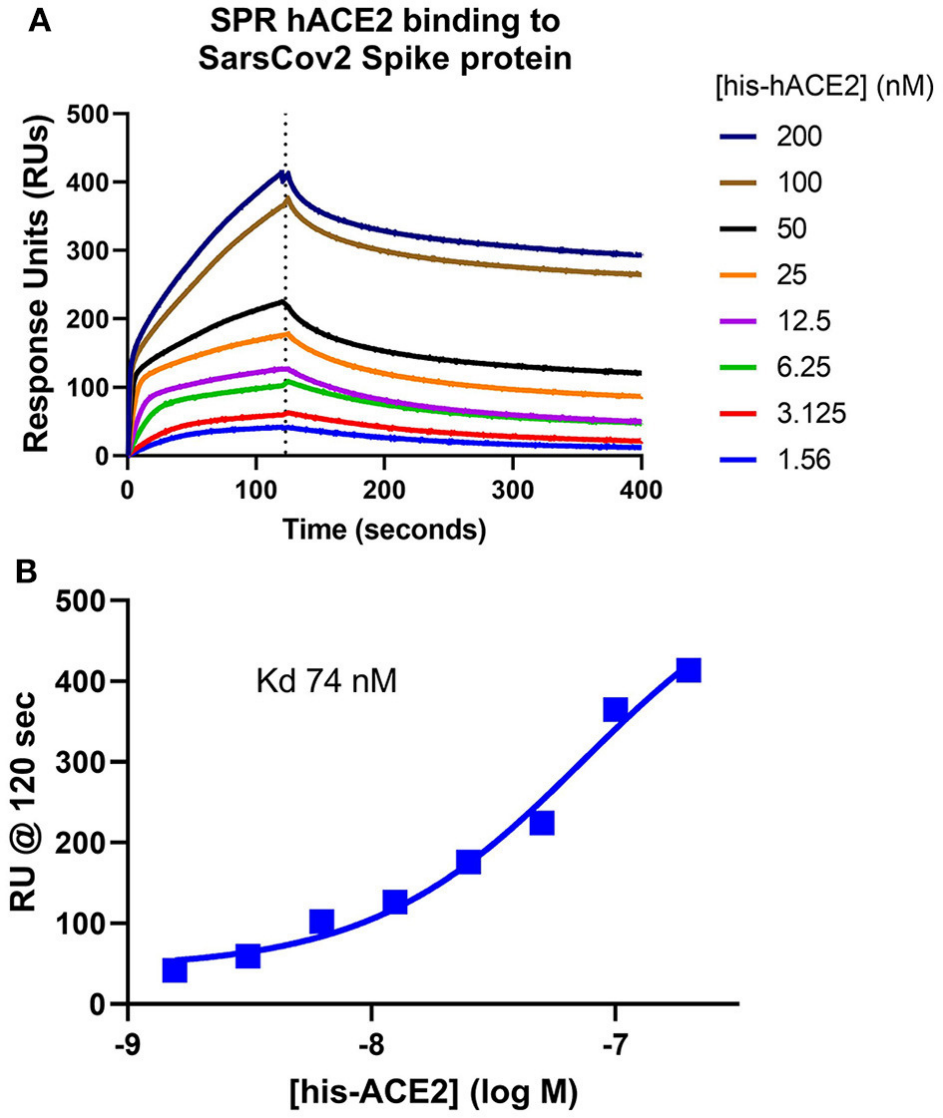
Surface Plasmon Resonance (SPR) determination of soluble ACE-2 binding affinity to SARS-CoV-2 spike protein. **(A)** Recombinant human ACE-2 (rhACE-2, Acrobiosystems), was analyzed by SPR assay for binding to a recombinant SARS-CoV-2 spike protein receptor binding domain (RBD, Acrobiosystems) immobilized in the flow chamber. As shown, increasing concentrations of the soluble rhACE-2 (in nM) showed proportional increases in binding to immobilized spike protein RBD. **(B)** These data allowed estimation of kD ~74 nM.

**Figure 2:**
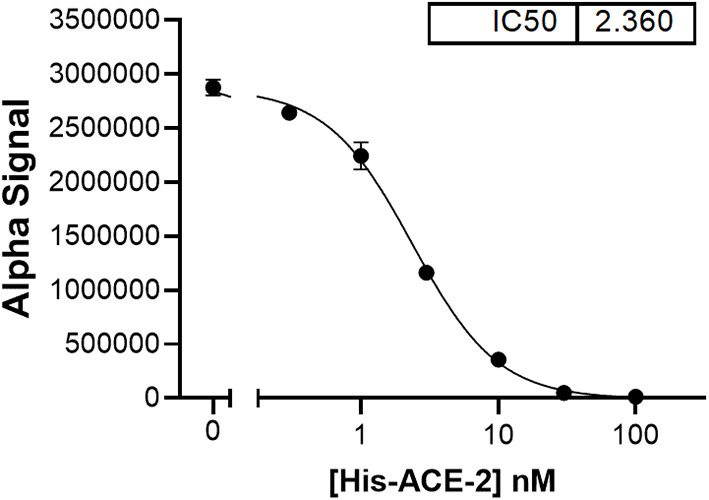
In a bead-based assay system [AlphaLISA proximity assay, (32)], which uses both rhACE-2 and spike RBD immobilized to beads, soluble rhACE-2 added to the assay buffer showed concentration-dependent inhibition of bead-bound spike RBD binding to bead-bound rhACE-2, with an IC50 ~2.4 nM.

The assumption that soluble ACE-2 binds SARS-CoV-2 is central to the hypothesis of autoimmunity to ACE-2. To begin making direct measurements to test this assumption, a preparation of recombinant human ACE-2 (rhACE-2, Acrobiosystems, amino acids 18-740) that is of similar length to that of soluble ACE-2 [18-708, (30)] was analyzed by SPR (surface plasmon resonance) assay for binding to a recombinant SARS-CoV-2 spike protein receptor binding domain (RBD, amino acids 321-591, Acrobiosystems). As shown in Figure 1A, increasing concentrations of rhACE-2 showed proportional increases in binding to the immobilized SARS-2 protein spike RBD. This enabled estimation of a binding affinity (kD) of ~74 nM (Figure 1B), which is in the range predicted by modeling studies and is greater than the binding affinity of SARS-CoV spike protein to ACE-2 (31). Figure 2 shows concentration-dependent inhibition of immobilized rhACE-2 binding to immobilized recombinant spike RBD, in this case determined by a bead-based Alpha-Assay (32), by soluble rhACE-2 added to the bead-based assay system at the concentrations indicated. Thus, the data in Figures 1, 2 strongly support the theory that a complex of SARS-CoV-2 and soluble ACE-2 is formed and circulating in the blood of infected patients.

Exploring differences in ACE-2 binding with the virus has not been straightforward, with some research suggesting similar binding affinities to SARS-COV (33) and other research inferring stronger complexes (34). The primary difference is the mutation in the spike protein which causes a 10-fold increase in the binding of SARS-COV-2 over that of SARS-CoV (31). Further, the same authors used cryo-EM studies to show that binding of ACE-2 to SARS-CoV-2 spike protein induces conformational changes in both proteins, which we postulate may form new epitopes that provide targets for autoantibody formation (35). This plays an important role in the hypothesis of autoimmunity.

There appears to be genetic polymorphism of the ACE-2 with increased risk of specific comorbidities—hypertension, cardiovascular disease, and diabetes (36, 37). The impact of allelic variants was reviewed in a computerized model and it was demonstrated that it is likely that some variations of ACE-2 will bind more tightly to the SARS-COV-2 spike protein (38). The current hypothesis of autoimmunity postulates that higher levels of soluble ACE-2, or augmented conformational binding to the spike protein, increases the probability that the combined entity will be processed by an antigen-presenting cell as part of the virus. This may lead to antibody production against ACE-2, which triggers Type 2 and 3 hypersensitivity responses, and Type 4 cellular immune targeting after the viral particles with attached soluble ACE-2 are processed by antigen-presenting cells.

Although most infectious diseases target both ends of the age spectrum because of either poorly-developed or impaired immune responses, COVID-19 disproportionately impacts the elderly. Soluble ACE-2 can explain the paradox of high mortality in the elderly without a similar raised infant mortality rate. Elevated levels of soluble ACE-2 have been noted in comorbidities associated with higher mortality in COVID-19 (39). There are undetectable levels in the serum of healthy individuals (40) and a correlation exists between the occurrence of soluble ACE-2 and an individual's age (41). Recent research has indicated that soluble ACE-2 is the most significant risk factor for cardiometabolic mortality and could be relevant in COVID-19 (42).

## Summary of Principle of Autoimmunity

It has been proposed that autoimmunity to ACE-2 (43) and ACE (2) is triggered by the viral infection in SARS-COV-2. It was postulated that in people with high levels of soluble ACE-2, the viral spike protein binding tightly together with ACE-2 could be endocytosed by macrophages which would function as antigen-presenting cells. The whole viral protein could be proteolytically cleaved, including ACE-2, tightly attached to the viral spike protein, possibly producing new epitopes for antibody generation. ACE-2 has a 42% homology with ACE, which could mean that autoantibodies that were formed against ACE-2 might cross- react with ACE as well. In this circumstance, the antibodies would target both ACE and ACE-2 cellular attached enzymes, leading to severe inflammation throughout the body, especially on lung endothelial cells. Consistent with this hypothesis, very recent data documented higher levels of soluble ACE-2 in the serum of critically ill CoVid-19 patients (44).

## Types of Hypersensitivity Immune Responses

There are four types of immune responses in the body, with the first three being antibody dependent (45) and the fourth being cellular (46).

### Type 1 Hypersensitivity

This is the typical anaphylaxis response to an external antigen after the body has been previously sensitized (47). This response is typically mediated by IgE after pre-sensitization to an antigen. The most important examples are that of pollen and nut allergies. In the context of autoimmunity to ACE-2, this is not considered to be particularly relevant as no significant difference with IgE was noted in COVID-19 (48).

### Type 2 Hypersensitivity

This represents the production of IgM or IgG antibodies to cellular or extracellular matrix proteins. An example of the extracellular antibody response is seen in Goodpasture's syndrome where Type 4 collagen is the antigen (49) which subsequently activates complement and the phagocytic system with associated kidney damage. Another example is immune thrombocytopenia which demonstrates a cellular immune response to platelets (50) with subsequent platelet destruction by this antibody response. In this case, the antigen is on the surface of platelets and the antibody will target the platelets with complement activation and increased phagocytosis. This is likely to be an early type of immune response in SARS-COV-2, with IgM produced against ACE and ACE-2, primarily targeting the endothelial blood vessels in the lung and small intestines. This was also demonstrated with specific B cell activation in the serum of patients with COVID-19 along a similar pattern to Systemic Lupus Erythematosus (51).

### Type 3 Hypersensitivity

The most common example is serum sickness where antigen and antibody complexes are formed in the blood and become deposited in tissues with associated inflammation (52). There is typically a systemic vasculitic response with endothelial swelling, inflammatory infiltrates, and fibrinoid necrosis of the arterial wall (53). This may be an early response in SARS-COV-2, with immune complexes deposited in the liver and lungs. In some of the limited autopsies in COVID-19, core lung biopsies revealed fibrinoid necrosis within pulmonary vessels (54), pathognomonic of Type III hypersensitivity.

### Type 4 Hypersensitivity

In this form of immune response, the antigen triggers the activation of CD8 lymphocytes which target cells that are infected with virus. This is an essential aspect of immune regulation as it allows the immune system to target virally-infected cells selectively (46). An early immune response to the viral complex with ACE-2 would also stimulate CD8 lymphocytes to target ACE-2 and, potentially, ACE. This is also likely to create immune depletion of lymphocytes as the immune system becomes overwhelmed. To date, the characteristic Type 4 hypersensitivity responses observed in COVID-19 include perivascular T-cell infiltrates (55, 56) with evidence of distal organ involvement in the adrenals (57) and giant cell pathology (58, 59).

The principle of autoimmunity can therefore explain the cytokine storm. It is likely to be a combination of three types of immune hypersensitivity—Types 2, 3, and 4. This condition has not previously been described in any other disease.

## Ferritin in COVID-19

Elevated levels of ferritin are a significant observation in COVID-19, with an increased risk of mortality (60). This hyperferritinemia is much worse than the typical liver function abnormalities that are also noted early in the course of the disease (61). Elevation of serum ferritin is a pattern in severe viral infections as well as septic shock (62). Whilst this association is observed, it is unclear if elevated ferritin is the cause of inflammation (63). Ferritin has been shown to have immune modulating functions (64, 65) and is also elevated in several autoimmune conditions (66). Significantly elevated serum ferritin levels also occur in hemochromatosis, a genetic disease, without a severe inflammatory response (67). This suggests that elevated ferritin, on its own, may not be a direct mediator of cellular damage. The presence of autoantibodies to hepatocytes and gastric chief cells, unique to serum from COVID-19 patients, is strongly suggestive of a Type II autoimmune response (68).

## Proposed Timeline of Symptoms

The following timeline and associated symptoms are based on the hypothesis of autoimmunity as the primary cause of disease:

Approximately 70% of people with COVID-19 are asymptomatic (69). This raises the question about whether the symptoms are linked primarily to the viral infection or to an autoimmune response. Based on this autoimmune theory, all symptoms, from the mild to more severe, can be positioned along the autoimmune spectrum.

- **Fever and mild coryzal symptoms** occur in 43.8%/50% on admission (70) and represents the immune viremic phase which is typically of relatively short duration (20, 71). It could be assumed that only viremia would cause these symptoms as an upper airway infection tends to be asymptomatic or have mild coryzal symptoms. The viremic phase occurs when viral particles are in the bloodstream and represents the starting point of symptoms. Those people with elevated levels of soluble ACE-2 or with genetic variations in the binding of the spike protein could be most prone to form IgM antibodies to ACE-2.
- **Mild shortness of breath with cough** could be triggered by IgM at day 3–4, targeting the endothelial-attached ACE and ACE-2 leading to Type 2 and 3 hypersensitivity. IgM could then cause endothelial damage in the lungs with associated shortness of breath secondary to pulmonary vascular leakage as evidenced by bibasal consolidation on the chest x-ray. There could also be microthrombi in the lung vessels.
- **Abnormal liver function tests** (“LFTs”) on admission to hospital occur in over half of patients (72) and may relate to Type 3 hypersensitivity, with immune complexes being deposited in the liver and subsequent hepatic inflammation. The IgM antibodies may bind to ACE-2 surface receptors on enterocytes in the small intestine and a proportion of these complexes are shed into the portal circulation. These immune complexes may then become lodged in the liver if the hepatic reticuloendothelial system becomes overwhelmed, and cause a mild hepatitis as evidenced by the abnormal LFTs early in the disease. There is no specific immune targeting in this case; the ACE-2 receptors are located in the small intestine and the complexes of ACE-2 plus IgM have to traverse the portal system. The observation of elevated serum ferritin is likely to be related to increased release from hepatocytes as part of the inflammatory response (66, 73).
- **Lymphopenia** could result from immune exhaustion in Type 4 hypersensitivity at day 7–14 (74). Once the viremia has occurred and soluble ACE-2 has become part of the immune response, CD8 lymphocytes are sensitized to target the lung, heart, and kidney due to overlap between ACE and ACE-2. The large number of antigens being targeted causes rapid lymphocyte exhaustion and lymphopenia (75).
- **CXR infiltrates** occur in 41% of positive COVID-19 admissions (76) and could indicate early lung damage at day 7–10. It involves Type 2, 3, and 4 hypersensitivity responses and may explain why this organ is primarily affected in mortality. Initially this is an IgM antibody response, but the pentameric structure of IgM may limit the number of ACE and ACE-2 receptors that can be targeted. Worsening of lung inflammation at day 11 could be linked to formation of IgG antibodies against ACE and ACE-2. Lung endothelial damage increases pulmonary vascular leakage with loss of albumin in the tissues and associated hypoalbuminemia (77).
- **Renal failure** occurs on admission in 14.4% of patients (78) as IgM could initially target ACE and ACE-2 receptors in the kidney leading to Type 2 hypersensitivity with involvement of renal tubular cells (79). There is no ACE in the glomerulus, but ACE-2 is located on the podocytes of the basement membrane in the glomerulus. Nephritis is not commonly a part of SARS-COV-2 (24).
- **Acute Respiratory Distress Syndrome (“ARDS”)** represents a severe deterioration in lung function possibly caused by formation of IgG antibodies at day 11 (80), worsening the Type 2 and 3 hypersensitivity response and leading to the cytokine storm. Clonal expansion of plasma cells with high levels of IgG to ACE-2 could increase lung and kidney inflammation. Persistent severe lung inflammation initiates fibroblast expansion, and hyaluronic acid is part of the immune response (81) which may contribute to lung fibrosis.
- **Kawasaki-like disease in children** (82) is likely to be Type 3 serum sickness-related vasculitis with fibrinoid necrosis in the arterial vessel walls.
- **Taste abnormalities** are possibly related to the targeting of ACE-2 receptors on taste buds (83) by IgA antibodies to ACE-2.
- **Myocarditis** is known to occur as an autoimmune response to viral infection (84). In COVID-19, myocarditis (85) may be triggered by Type 2 hypersensitivity to ACE and ACE-2 receptors on the myocardium.
- **Stroke** could be secondary to myocarditis combined with increased levels of clotting factors released from the liver secondary to immune-mediated hepatitis. Although a number of patients may have had a stroke during the COVID-19 infection, it is not clear if this represents a higher risk (86).

## Conclusion

The autoimmune hypothesis appears to explain a significant proportion of the symptoms in SARS-COV-2-induced organ damage, along the lines of similar autoimmune diseases. In the vast majority of people affected, the disease is mild or asymptomatic. Identification of significant risk factors including elevated serum ACE-2 is critical as it would allow vaccination to target the most vulnerable population. This theory of autoimmunity in SARS-COV-2 has been reinforced by the recent RECOVERY Trial (87) showing the benefit of the steroid dexamethasone for immunosuppression. Additionally, high-dose methylprednisolone has also been proposed as a rescue, second-line treatment for patients who did not respond well to tocilizumab (88). In both treatment methods, there appears to be a benefit which would support the hypothesis of immune dysregulation due to autoimmunity to ACE and ACE-2.

In the time since the original submission of this manuscript, autoimmunity to COVID-19 has now been confirmed, as autoantibodies to ACE-2 have been demonstrated in the serum of individuals with severe disease (89). Autoantibodies to Type I interferons have also been characterized in severe COVID-19 (90) and may represent a bystander effect, in keeping with our primary hypothesis of autoimmunity to ACE-2. Whilst these studies are supportive of the theory of autoimmunity, further research will have to be conducted to demonstrate conclusively that autoantibodies are the cause of the organ damage observed in COVID-19. We invite the research community to help explore these hypotheses for the betterment of clinical strategies for treating individuals suffering from this disease.

## Author Contributions

PM conceived and drafted the original manuscript. TD and RN conceived and performed protein-protein interaction experiments to obtain the original data. All authors edited, revised and approved the final manuscript.

## Conflict of Interest

The authors declare that the research was conducted in the absence of any commercial or financial relationships that could be construed as a potential conflict of interest.
